# Surgical Treatment of Patellofemoral Instability in Children: Lessons From a 10-Year Single-Center Experience

**DOI:** 10.7759/cureus.115128

**Published:** 2026-08-24

**Authors:** Ismail Benomar, Loubna Aqqaoui, Hidaya Zitan, Houda Oubejja, Fouad Ettayebi, Sarah Hosni, Sidi Zouhair Fellous El Alami, Tarik El Madhi

**Affiliations:** 1 Department of Pediatric Surgical Emergency, Children’s Hospital of Rabat, Ibn Sina University Hospital Center, Faculty of Medicine and Pharmacy, Mohammed V University, Rabat, MAR; 2 Laboratory of Biology and Health, Faculty of Science, Ibn Tofail University, Rabat, MAR; 3 Department of Pediatric Trauma and Orthopedic Surgery, Children’s Hospital of Rabat, Ibn Sina University Hospital Center, Faculty of Medicine and Pharmacy, Mohammed V University, Rabat, MAR; 4 Department of Pediatric Trauma and Orthopedic Surgery, Children's Hospital of Rabat, Ibn Sina University Hospital Center, Faculty of Medicine and Pharmacy, Mohammed V University, Rabat, MAR

**Keywords:** child, medial patellofemoral ligament (mpfl), patellar dislocation, patellofemoral instability, pediatric orthopedic surgery, trochlear dysplasia

## Abstract

Background: Patellofemoral instability (PFI) in children is a complex, multifactorial condition and a major challenge in pediatric orthopedics, yet no epidemiological data exist for the Moroccan pediatric population.

Methods: We retrospectively reviewed 20 children (25 knees) treated for patellar instability over a 10-year period (2015-2025) in the pediatric trauma and orthopedic surgery department of the Children's Hospital of Rabat. Epidemiological, clinical, radiological, and therapeutic data were analyzed and compared with the international literature. Most patients were treated with a modified Insall procedure combining proximal realignment, a Roux-Goldthwait patellar tendon transfer, and medial patellofemoral ligament (MPFL) reconstruction.

Results: Mean age at surgery was 10 years (range: 5-17), with a female predominance of 12/20 patients (60%). Bilateral involvement was present in 5/20 patients (25%), and no familial cases were recorded. Recurrent dislocation was more common (15/25 knees, 60%) than permanent dislocation (10/25 knees, 40%). Imaging confirmed the multifactorial anatomical basis of the disease, with trochlear dysplasia, patella alta, and an increased tibial tubercle-trochlear groove distance frequently identified. After a mean follow-up of five years, excellent results were obtained in 23/25 knees (92%), with 2/25 knees (8%) presenting recurrence and 1/25 knees (4%) presenting a wound infection with a favorable outcome.

Conclusion: An individualized, "menu à la carte" surgical strategy adapted to each child's anatomical profile and growth potential provides satisfactory functional and anatomical outcomes in pediatric PFI, with results comparable to or better than those reported in the international literature.

## Introduction

Patellofemoral instability (PFI) in children encompasses a spectrum of conditions ranging from mild patellar apprehension to permanent, irreducible dislocation and includes first-time traumatic dislocation, recurrent dislocation, and habitual or congenital dislocation [1]. It is a frequent cause of anterior knee pain and functional disability in the growing child, and its management remains one of the more debated areas of pediatric orthopedic surgery.

Population-based studies estimate the overall incidence of patellar dislocation at approximately six per 100,000 person-years, rising to nearly 29 per 100,000 among adolescents aged 10-17 years [2]. A recent analysis of the US National Electronic Injury Surveillance System (NEISS) identified 208,673 pediatric emergency department presentations for patellar instability between 2001 and 2020, with a significant annual increase of approximately 256 cases per year [3], while a separate nationwide database reported an overall incidence rising from 2.58 to 3.0 per 100,000 person-years over the same period, with the sharpest increase in patients aged 10-19 years [4]. To date, no epidemiological data on this condition are available for the pediatric population in Morocco.

The stability of the patellofemoral joint depends on a balance between static and dynamic restraints: trochlear morphology and depth, patellar height, the rotational and coronal alignment of the lower limb (reflected by the tibial tuberosity-trochlear groove (TT-TG) distance), the integrity of the medial patellofemoral ligament (MPFL), and the balance of the quadriceps muscle [1]. Failure of one or more of these factors - most commonly trochlear dysplasia, patella alta, an increased TT-TG distance, and quadriceps dysplasia - predisposes to instability, which explains the markedly heterogeneous clinical presentations encountered in practice.

Management of PFI in the growing child is further complicated by the presence of open growth plates, which constrain the choice of surgical technique, and by the existence of congenital and habitual forms that are specific to the pediatric population and are not encountered in adults. Diagnosis relies on a thorough clinical examination complemented by standardized radiographic views, computed tomography (CT) and magnetic resonance imaging (MRI), which together allow identification of the anatomical risk factors and associated lesions that guide treatment. While conservative management retains an important role in first-time dislocation without major anatomical risk factors, an individualized surgical strategy - tailored to each anomaly identified, following a "menu à la carte" philosophy - is increasingly favored for recurrent or permanent instability.

The aim of this study was to describe the epidemiological, clinical, radiological, and therapeutic characteristics of pediatric PFI in a Moroccan population, to report the functional and anatomical outcomes of a modified surgical technique combining the Insall proximal realignment, a Roux-Goldthwait patellar tendon transfer, and MPFL reconstruction, and to compare these findings with the international literature.

## Materials and methods

This retrospective study was conducted in the Department of Pediatric Trauma and Orthopedic Surgery and the Department of Pediatric Surgical Emergency at the Children's Hospital of Rabat over a 10-year period (2015-2025).

Inclusion criteria were children under 18 years of age presenting with clinical and radiological evidence of PFI (recurrent dislocation, permanent/habitual dislocation, or subluxation), who underwent surgical treatment during the study period, and whose medical records contained complete clinical, radiological, and follow-up data.

Exclusion criteria were incomplete medical records (missing clinical, radiological, or follow-up data), patellar instability secondary to acute traumatic injury without underlying anatomical predisposition, and patients lost to follow-up before a minimum postoperative evaluation.

Twenty-two medical records were initially identified; two were excluded because of incomplete data, leaving 20 patients (25 operated knees) available for analysis, with a mean follow-up of five years.

The primary outcome of this study was the rate of surgical success, defined as the absence of recurrent patellar dislocation at last follow-up. Secondary outcomes included functional and anatomical results according to the Insall clinical criteria, postoperative complications (including wound infection), and correction of the underlying anatomical risk factors (trochlear dysplasia, patella alta, increased tibial tubercle-trochlear groove distance) as assessed on postoperative imaging.

Clinical, radiological, and surgical data were extracted from the medical records and compared with data from the international literature to assess the concordance of our results and the relevance of the therapeutic approaches used. The parameters studied included age at treatment, sex, number of joints operated, duration of symptoms before consultation, clinical presentation according to the form of instability, findings on standard radiographs (anteroposterior, lateral at 30° of flexion, and axial at 30° of flexion views), CT and MRI findings, and functional and anatomical outcomes after surgical treatment.

Surgical technique

The procedure used in the majority of cases was a modified Insall procedure [5], combining three complementary steps performed through a single medial parapatellar approach: (1) proximal realignment as originally described by Insall, consisting of a lateral retinacular release combined with medial advancement and plication of the vastus medialis obliquus to restore dynamic medial restraint; (2) a Roux-Goldthwait transfer, in which the lateral third or half of the patellar tendon is detached from the tibial tubercle and rerouted medially, then sutured under the medial half of the tendon or to the periosteum, to correct the extensor mechanism alignment without disturbing the tibial tuberosity apophysis in skeletally immature patients; and (3) reconstruction of the MPFL. In children with recurrent instability, surgery was preceded by a minimum of six months of supervised physiotherapy focused on quadriceps strengthening.

Functional and anatomical outcomes were assessed using the Insall clinical criteria, originally described for the evaluation of patellofemoral disorders [6], which classify results into four categories based on residual pain, knee stability, and return to activity level: (1) Excellent: no pain, no instability, full return to pre-injury activity level; (2) Good: occasional mild discomfort, no instability, slight limitation of activity; (3) Fair: intermittent pain and/or subjective instability, moderate limitation of activity; and (4) Poor: persistent pain, recurrent instability, or need for further surgery. Surgical success was defined as the absence of recurrent dislocation at last follow-up.

## Results

Patients' ages at surgery ranged from 5 to 17 years, with a mean of 10 years. Table 1 summarizes the age distribution.

**Table 1:** Age distribution at the time of surgery (n = 20 patients; mean age 10 years, range 5–17 years).

Age at surgery	0–5 years	6–10 years	>10 years
Number of patients	5	6	9
Percentage	25%	30%	45%

A clear female predominance was observed, with girls accounting for 60% of the series. No familial cases of patellar instability were identified. Bilateral involvement was present in 25% of patients, while 75% had unilateral disease (Table 2).

**Table 2:** Laterality of involvement (n = 20 patients).

Laterality	Bilateral	Unilateral
Number of patients	5	15
Percentage	25%	75%

The main presenting complaints were recurrent falls, anterior knee pain, gait instability with limping, a sensation of insecurity on weight-bearing, reduced physical activity, and, in some cases, knee locking with an inability to walk. The mean age at symptom onset was nine years, ranging from the acquisition of independent walking to adolescence.

Two clinical forms were identified: permanent patellar dislocation, observed in eight knees (40%), and recurrent patellar dislocation, observed in 12 knees (60%). In the permanent form, associated lower-limb malalignment included genu valgum (two cases) and genu recurvatum (one case); a preceding traumatic event was reported in three cases. On examination, the patella was unstable and laterally displaced in both flexion and extension in six knees, a positive Smillie sign was present in four patients, patellar hypermobility in three cases, a patellar shock sign in one knee, unilateral quadriceps amyotrophy in two children, and planovalgus feet in three patients. In the recurrent form, genu valgum was present in three patients, nine children reported dislocation episodes associated with gait instability, a positive Smillie sign was found in two cases, and patellar hypermobility was present in all patients of this subgroup.

Standard radiographic assessment (anteroposterior and lateral views at 30° of flexion) was performed systematically, complemented by an axial view at 30° of flexion in three patients. CT was performed in five patients and MRI in six patients, all with the lower limb at rest and without active quadriceps contraction.

The trochlear opening angle exceeded 150° in all 10 knees in which it was measured. The TT-TG distance, measured in five knees, was greater than 15 mm in two knees and less than 15 mm in the remaining three. Lateral trochlear inclination, measured in four knees, exceeded 15° in all of them. Additional CT/MRI findings included signs of trochlear dysplasia in six knees, MPFL rupture in three knees, minor meniscal lesions in one knee, patella alta in two patients (including one case of bipartite patella), intra-articular calcified bodies in one knee, patellar dysplasia in five cases, and lower-limb length discrepancy in one patient.

A total of 25 knees were operated on. All patients with recurrent instability who underwent surgery had received at least six months of physiotherapy beforehand. The interval between symptom onset and surgery ranged from five months to three years (mean 12 months). In the great majority of cases, the technique used was the modified Insall procedure described above.

Postoperative follow-up ranged from five months to six years (mean 19 months). At last follow-up, results were excellent in 23 knees, while two knees presented a recurrence of instability, corresponding to an overall success rate of 92% (Table 3). One case of surgical-site infection with wound dehiscence was recorded, with a favorable outcome after appropriate management.

**Table 3:** Surgical outcomes at last follow-up (mean 19 months; range five months to six years).

Outcome	Knees	Percentage
Excellent (no recurrence)	23	92%
Recurrence of instability	2	8%
Total operated knees	25	100%

## Discussion

Patellar instability accounts for 2-3% of all knee complaints [7], and its incidence rises sharply during adolescence, reaching about 29 per 100,000 among 10-17-year-olds [8]. Large administrative-database studies confirm a steadily increasing incidence over the past two decades, particularly among adolescents, with roughly two-thirds of dislocations related to sport [4]. The mean age of our patients (10 years) was lower than in most published pediatric and adolescent series - for instance, the Mayo Clinic cohort reported a mean age of 14.9 years [9], and a large single-center US cohort a median age of 15.2 years [10] - a difference explained by the relatively high proportion of congenital and habitual forms in our series, which typically present earlier in childhood.

A female predominance is consistently reported in the literature, particularly among adolescents [10], and is generally attributed to sex-related anatomical differences (increased trochlear dysplasia and patella alta, greater Q angle, and greater generalized ligamentous laxity) [11], although some authors found no significant effect of female sex on recurrence risk after a first dislocation [12].

Girls also appear to more frequently require surgical treatment and to be at higher risk of postoperative complications and less favorable functional outcomes after MPFL reconstruction [10]. Our series confirms this female predominance, with a 60% proportion of girls, closely matching the 60.8% reported in a large national pediatric hospitalization database [13] and the demographic profile of the prospective multicenter JUPITER cohort [14].

Bilateral involvement, found in 25% of our patients, is a recognized feature of pediatric PFI; a dedicated study of 32 patients with bilateral disease found that patients typically presented at least two, and often three or four, anatomical risk factors per knee, with marked symmetry between sides [15]. No familial cases were identified in our cohort, whereas a 2023 systematic review found a familial association in 17 of the studies reviewed, with up to a fourfold increased risk of contralateral dislocation in patients with a positive family history [16], and several reports describing multigenerational transmission and associated genetic syndromes [17]. All dislocations in our series were lateral, consistent with the literature, in which medial dislocation is exceptional and most often reported as an iatrogenic complication of excessive lateral release [17].

The clinical spectrum observed in our series - ranging from permanent, irreducible dislocation to recurrent episodes triggered by minimal trauma - mirrors the two principal phenotypes described in children, each carrying distinct implications for management and prognosis [2]. The high frequency of associated coronal-plane misalignment (genu valgum) and of positive apprehension and hypermobility signs in our cohort is consistent with the recognized contribution of both bony and ligamentous factors to the clinical presentation.

Imaging in our series confirmed the multifactorial anatomical basis of instability, with a high prevalence of trochlear dysplasia (open trochlear angle in all measured knees, lateral trochlear inclination >15° in all measured knees), patella alta, and an increased TT-TG distance in a subset of patients. These findings are in keeping with the recognized principal risk factors for both first-time and recurrent dislocation, which act cumulatively rather than in isolation [16]. The relatively small number of knees in which each radiological parameter could be formally measured is a limitation of our imaging protocol, discussed further below.

Isolated lateral retinacular release, once popular, is now recognized as ineffective when used alone, since it does not achieve effective medial recentring of the patella; despite initially high short-term satisfaction, functional results deteriorate significantly over the medium and long term [18]. When correctly indicated as part of a combined procedure, however, reported success rates range from 70% to 85%, with a complication rate around 7% mainly related to tourniquet use and prolonged drainage [19].

For congenital and habitual dislocation in flexion, extensive quadriceps release according to Judet, combined with individualized "à la carte" realignment procedures, has shown good results, with a complication rate of 11% and a recurrence rate of 8% at a mean 6.5-year follow-up, together with excellent functional scores (mean Kujala score of 93%) [20]. In children with Down syndrome, a standardized protocol combining lateral release, MPFL reconstruction with quadriceps tendon autograft, a Roux-Goldthwait procedure and, when necessary, V-Y quadricepsplasty achieved a stable, non-recurrent patella in all patients, with mean Kujala scores improving from 52.6 to 92.2 [21].

Comparative data on the Insall proximal realignment itself are more contrasted. At a mean nine-year follow-up, isolated MPFL reconstruction was associated with markedly less radiological patellofemoral osteoarthritis (6.7% versus 60%) and fewer reoperations (0% versus 40%) than the Insall procedure in adolescents, despite similar functional scores [22]. A Norwegian cohort of 46 patients treated by Insall proximal realignment, sometimes combined with tibial tuberosity osteotomy, reported acceptable patient-reported outcomes (mean Kujala score 75.4/100) and a limited recurrence rate (10.6%) at 6.6 years, but a marked increase (about 15%) in radiological osteoarthritis between the pre- and postoperative periods [23]. A German series of 30 knees treated with a modified Roux-Goldthwait technique reported excellent results according to Insall's criteria in 26 knees, with quadriceps strength recovering to 80-90% of the contralateral side, while a historical 30-year follow-up study of the Roux technique found that, at very long term, clinical and radiographic outcomes of operated and non-operated contralateral knees became similar, suggesting that a single non-individualized procedure may not address every underlying cause of instability [24].

Against this background, the 92% success rate obtained in our series with the modified Insall-Roux-Goldthwait-MPFL combination compares favorably with most published outcomes for isolated bony realignment procedures and is closer to the results typically reported after ligamentous reconstruction. This likely reflects the individualized, combined nature of our technique, which associates soft-tissue medialization with MPFL reconstruction rather than relying on bony realignment alone.

For patients with severe trochlear dysplasia, the literature indicates that adding a trochleoplasty to MPFL reconstruction reduces the redislocation rate compared with isolated MPFL reconstruction, particularly in high-grade dysplasia (with redislocation rates as low as 0.9% versus 16.2% without trochleoplasty in one series of 459 knees), without necessarily reducing recurrence rates in low-grade dysplasia, where the two strategies perform similarly [25]. A recent systematic review confirms that MPFL reconstruction alone reliably reduces redislocation rates to below 5% in well-selected patients, while combined procedures (tibial tuberosity osteotomy, trochleoplasty) are reserved for cases with an increased TT-TG distance or severe trochlear dysplasia, with low complication rates (2-7%) when growth-plate-sparing techniques are used in children [26]. Reported patient satisfaction after trochleoplasty ranges from 67% to 91%, with more than 75% of young patients returning to sport [27], and long-term series report persistent satisfaction in around 81% of patients at 15 years, although residual instability sensation and intermittent pain may persist in a minority [28]. None of our patients required trochleoplasty, reflecting the relatively lower proportion of high-grade trochlear dysplasia in our cohort.

Persistent postoperative pain is relatively common after procedures addressing severe patellofemoral disease, particularly in patients with pre-existing cartilage lesions or previous failed surgery [29]. Reported outcomes after trochleoplasty specifically range from improvement in about half of patients to worsening in around a third at intermediate follow-up, and pain trajectories at longer follow-up remain variable across series [30]. In our series, complications were limited to a single case of superficial wound infection with dehiscence, which resolved favorably, and no cases of postoperative stiffness, growth disturbance, or symptomatic osteoarthritis were recorded during the follow-up period, likely reflecting both the relatively short mean follow-up (19 months) and the absence of bony trochlear procedures in our cohort.

This study has several limitations inherent to its design. The retrospective, single-center design and the small sample size (20 patients, 25 knees) limit the statistical power of comparisons and the generalizability of our findings. Several radiological parameters (TA-GT distance, lateral trochlear inclination) were measured in only a subset of patients, reflecting heterogeneity in the imaging protocols used over the 10-year study period, and may therefore underestimate their true prevalence in this population. The mean postoperative follow-up of 19 months, while sufficient to assess early functional and anatomical outcomes, is too short to reliably detect late complications such as osteoarthritis progression, which typically emerge after several years, as illustrated by several of the long-term series discussed above. Finally, the absence of a standardized, validated functional outcome score (such as the Kujala or IKDC score) limits direct comparison with more recent international series.

## Conclusions

PFI in children is a frequent, multifactorial condition whose functional consequences can be substantial in the absence of appropriate management. Its pathophysiology, combining anatomical abnormalities, biomechanical disturbances, and ligamentous insufficiency, accounts for the diversity of clinical presentations and the difficulty of its treatment. Diagnosis relies primarily on rigorous clinical assessment, supplemented by precise radiological evaluation - increasingly reliant on MRI and CT - to identify anatomical risk factors and associated lesions. Management must be individualized for each patient according to age, growth potential, severity of instability, and associated anatomical abnormalities: while conservative treatment retains an important role in selected early forms, advances in surgical technique have significantly improved functional outcomes and reduced recurrence risk while respecting the specific anatomical constraints of the growing child. In our series, a modified surgical strategy combining the Insall proximal realignment, a Roux-Goldthwait patellar tendon transfer, and MPFL reconstruction achieved excellent functional and anatomical results in the large majority of patients, with a success rate of 92% and few complications. These results support the value of an individualized, "menu à la carte" surgical approach in this population, while underlining the need for further research, ideally multicentric and with longer follow-up, to further refine diagnostic and therapeutic strategies and to prevent long-term complications such as chronic instability and early patellofemoral osteoarthritis.
